# Peritoneal dialysis in Sichuan province of China – report from the Chinese National Renal Data System

**DOI:** 10.1080/0886022X.2018.1496933

**Published:** 2018-10-22

**Authors:** Changwei Wu, Xiuling Chen, Amanda Ying Wang, Jin Chen, Hui Gao, Guisen Li, Li Wang, Daqing Hong

**Affiliations:** a Renal Department and Nephrology Institute, Sichuan Provincial People’s Hospital, School of Medicine, University of Electronic Science and Technology of China, Chengdu, China;; b The George Institute for Global Health, University of Sydney, Sydney, Australia

**Keywords:** Clinical outcomes, peritoneal dialysis registry, peritonitis, retrospective study

## Abstract

**Background:** Peritoneal dialysis (PD) is one of the important treatment strategies for end stage renal disease (ESRD). In this study, we aimed to study the patients on PD of Sichuan province in the registry system and to explore the risk factors.

**Methods:** This was a retrospective study based on data from the Chinese National Renal Data System (CNRDS). The outcomes were prevalence and incidence of patients receiving PD, all-cause mortality, technical failure, end events and peritonitis.

**Results:** This study included 2654 patients between 1 January 2010 and 31 December 2016. From 2010 to 2016, despite there were increasing numbers of patients requiring PD. Primary and secondary glomerular diseases were the main causes of ESRD. Erythropoietin, iron and antihypertensive agents were the most commonly used medications in this cohort. 12.43% of patients died and the most important cause of death was cardiac events (30.30%). The incidences of peritonitis were 0.09, 0.16, 0.11, 0.09, 0.08, 0.12 and 0.06 per patient-year, respectively. The most common etiological agent of peritonitis was staphylococcus. We divided the patients into four groups according to the incident months of peritonitis. Compared with <20 months group, the level of calcium and platelet in >60 months group were higher, and the level of ferritin in >60 months group was lower.

**Conclusion:** Our results, representing the first largest report of peritoneal dialysis in the Southwest of China, indicated increasing numbers of patients receiving peritoneal dialysis, which will require need for medical resource.

## Introduction

Kidney disease is now recognized as a substantial worldwide public health problem [1–3]. Peritoneal dialysis is one of the important treatments for end stage renal disease (ESRD). At present, ∼11% of ESRD patients in the world are treated with peritoneal dialysis, which means that more than 272,000 patients receive peritoneal dialysis [4]. Among this population, maintaining the technical survival of PD is important for ESRD patients. Dialysis practice patterns and outcomes are important to maintain the technical success of the therapy and optimize patient outcomes [5]. Peritonitis is a major complication of PD and one of the most common causes of death and technique failure [6–8]. These varied greatly between centers, most commonly with smaller centers where lower cumulative numbers of patients experienced higher rates of technique failure [9,10]. Thus it is essential to establish a standard protocol to record the information of peritoneal dialysis across each center to improve both clinical care and also the quality of programmatic healthcare delivery.

The registry using advanced information technology creates and stores information, which can maintain uniformity of the information being stored and improve the quality. These registries are an integral part of quality control processes for renal replacement services and provide a tool for benchmarking of clinical outcomes within and between countries [11]. Many countries such as Brazil [12], French [13], Japan [14], Brunei [11], Iran [5], Australia and New Zealand [7,15] have already conducted the peritoneal dialysis registry. The Chinese National Renal Data System (CNRDS) was established in 2010 to collect data from patients undergoing peritoneal dialysis and hemodialysis in dialysis centers of China. There are nine domains of information including sociodemographic, clinical and laboratory characteristics, treatment modalities, medicine treatment, dialysis information, infection, complication and outcome.

In this study, we aimed to study the patients on peritoneal dialysis of Sichuan province in the registry, to analyze the prevalence and incidence of PD and to explore the risk factors of peritonitis and technical failure of PD.

## Subjects and methods

### Study population

All peritoneal dialysis patients in Sichuan province between 1 January 2010 and 31 December 2016 of the Chinese National Renal Data System (CNRDS) including 35 centers covering 13 areas of Sichuan province. The centers that had not collected data regularly or had missed more than half of data during the study period were excluded. Finally, total 33 centers were enrolled. Only patients having the baseline information and followed up regularly were included in this retrospective study.

### Outcomes

The primary outcomes were the prevalence and incidence of patients receiving PD in each calendar year. The end events included four situations: on dialysis, died, dropped out and rolled out. If patients were on peritoneal dialysis until 31 December 2016, the end time was 31 December 2016, otherwise the end time was the time of the outcome. Technique survival was defined as survived patients being able to continue PD. Patient survival was defined as patients that remained survived, excluding patients died of all-cause. To assess patients’ peritonitis, we described the peritonitis rate as number of episodes per patient-year. And we calculated the incident month on peritonitis of each patient: total months of follow-up/total frequency incident on peritonitis. Besides, the end time of peritonitis was the first time when incident peritonitis occurred.

### Data collection and cleaning

The individual characteristics extracted from the registry were age, gender, causes of ESRD and death, comorbidities, outcomes, the time of commencement of PD and outcomes, treatments used for peritoneal dialysis, the progress of peritonitis and the laboratory examinations. Unidentified data were obtained to keep confidentiality.

The baseline clinical data, the variety of treatment medicine, start time and end time of peritoneal dialysis, the causes of outcome and the time incident peritonitis of all patients enrolled in this study were summarized in Table 1. Because the fact that the information about first dialysis of some patients could not be traced back and the information deletion of some centers, the baseline clinical data was defined as the first clinical data that entered to the registry.

**Table 1.** The demographics of patients on peritoneal dialysis.

	Number	Percentage (%)
Men/female	1518/1133	57.26/42.74
Age (year)	53 ± 15^a^	
<20	15	0.58
20–40	517	19.92
40–60	1206	46.46
≥60	858	33.05
Hypertension	1662	62.62
Cardiac disease	1692	63.75
Comorbidities		
0	389	14.66
1	378	10.47
2	182	6.86
3	1033	38.92
≥4	672	25.32
Cause of ESRD		
Primary glomerular disease	1210	45.59
Secondary glomerular disease	649	24.45
Hereditary nephritis	27	1.02
Unclear	741	27.92
other	27	1.02

* Mean ± SD.

The cutoff of standard in laboratory examination was defined by the Blood Purification Standard Operating Procedure: Hemoglobin (HB) in 110–120 g L^−1^, Albumin (ALB) > 35 g L^−1^, Immunoreactive parathyroid hormone (IPTH) in 150–300 pg mL^−1^, Calcium (Ca) in 2.1–2.37 mmol L^−1^, Phosphorus (P) in 1.13–1.78 mmol L^−1^, Ferritin (FER) > 200 ng mL^−1^ and total endogenous creatinine clearance rate (CCR) > 50 mL min^−1^.

### Statistical analyses

The prevalence, demographics and clinical characteristics, treatment, causes to death and the incident months incident on peritonitis were summarized as descriptive statistics which were expressed as percentages, frequencies and means and SD. The effects of baseline clinical variable on outcome and peritonitis were conducted by Kaplan–Meier survival analysis. The Kruskal–Wallis H test was used to analyze the difference of baseline clinical variables and the non-normal distribution. We divide the incident months of peritonitis to four groups, <20, 20–40, 40–60 and 60 months, respectively. And differences of clinical variables in different groups were analyzed by Kruskal-Wallis H test. All analyses of this registry data were performed with SPSS 19.0 software version (SPSS, Chicago, IL). The strength was indicated by *p* values which <0.05 was considered statistically significant.

## Results

### Prevalent and incident peritoneal dialysis

**Figure 1.**
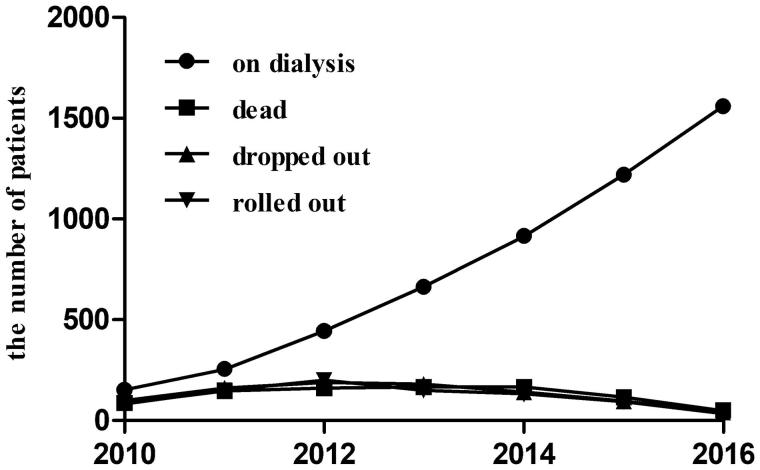
The prevalence of patients on peritoneal dialysis. From 2010 to 2016, the numbers of patients on dialysis were 154, 256, 446, 664, 916, 1220 and 1561, respectively. The numbers of patients who dead were 88, 148, 161, 165, 165, 116 and 50, respectively. The numbers of patients who dropped out were 101, 163, 190, 180, 142, 97 and 42, respectively. The number of patients who rolled out were 83, 149, 201, 150, 133, 94 and 36, respectively.

During the study period (2010–2016), a total of 2654 patients undergoing peritoneal dialysis were enrolled. Of these, 1623 (61.15%) patients were still on dialysis, 330 (12.43%) patients died, 330 (12.43%) patients dropped out from the study including 319 patients transferred to hemodialysis, and 371 (13.98%) patients rolled out including 148 patients receiving the kidney transplant and 233 patients withdrawn from the dialysis. From 2010 to 2016, the number of patients on dialysis was on the rise, while the number of patients who died, dropped out or rolled out were decreasing (Figure 1). In total, the percent of technique survival was 75.55%, and which of patient survival was 87.57%.

In this cohort, the mean follow-up period was 31.36 ± 25.90 months. In addition, the number of patients still on dialysis at 1, 3, 5 and 10 years were 1829 (71.81%), 911 (35.77%), 340 (13.35%) and 17(6.67%), respectively (Supplemental Figure 1).

### Demographics and clinical characteristics

The mean age of the patients was 53 ± 15 years, with only 87 (3.35%) patients being more than 80 years of age. In this cohort, 57.26% of peritoneal dialysis patients were men, 1662 (62.62%) patients had hypertension, and 1692 (63.75) patients had cardiovascular diseases. Primary glomerular diseases and secondary glomerular diseases were the major causes leading to ESRD, which accounted for 70.04% of this cohort. More than a half of patients had more than three comorbidities including hypertension, diabetes, cardiovascular diseases. The all detailed information about demographics and baseline clinical characteristics was showed in Tables 1 and 2.

**Table 2.** The clinical characteristics of patients on peritoneal dialysis.

	Mean ± SD	Number	Percentage (%)
SCr (µmol L^−1^)	875.00 ± 341.95		
BUN (mmol L^−1^)	27.11 ± 17.12		
Total Kt/V	2.12 ± 3.70		
Residual CCR (mL min^−1^)	32.55 ± 47.87		
HB (g L^−1^)	84.38 ± 21.14	2372	
<110		2112	89.04
110–120		123	5.19
≥120		137	5.78
FER (ng mL^−1^)	373.08 ± 460.06	1508	
≤200		636	42.18
>200		872	57.82
Alb (g L^−1^)	36.39 ± 6.11	2160	
≤35		846	39.17
>35		1314	60.83
iPTH (pg mL^−1^)	219.30 ± 298.01	1660	
<150		963	58.01
150–300		256	15.42
≥300		441	26.57
Ca (mmol L^−1^)	2.04 ± 0.34	2222	
<2.1		1250	56.26
2.1–2.37		758	34.11
≥2.37		214	9.63
P (mmol L^−1^)	1.82 ± 0.64	2207	
<1.13		223	10.10
1.13–1.78		931	42.18
≥1.78		1053	47.71
Total CCR (mL min^−1^)	32.55 ± 47.87	1754	
≤50		605	34.49
>50		1149	65.51

SCr: serum creatinine, BUN: blood urea nitrogen, HB: hemoglobin, FER: ferritin, ALB: albumin, iPTH: immunoreactive parathyroid hormone, CCR: endogenous creatinine clearance rate.

At baseline, the mean serum creatinine (SCr) and mean BUN were 875.00 ± 341.95 μmol L^−1^ and 27.11 ± 17.12 mmol L^−1^, respectively. A 39.17% of patients had hypoalbuminaemia (serum albumin <35 g L^−1^), 89.04% of patients were anemic (HB <110 g L^−1^) and almost a half of patients were hypocalcaemic and hyperphosphataemic.

### Concurrent treatments among peritoneal dialysis patients

Use of erythropoietin, antihypertensive drugs, iron, calcium agents, phophorus lowering medicine and Vitamin D used in PD patients were recorded into the registry, in which erythropoietin, iron and antihypertensive agents were the most commonly used medications in this cohort (Supplement Figure 2). About half of patients were treated with these three drugs.

**Figure 2.**
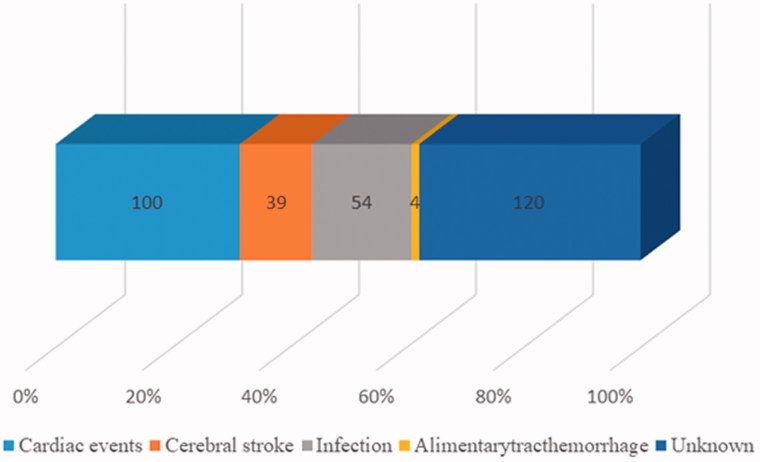
The causes of death. *X*-axis stood for the distribution of percentages, and the digital in the graph stood for the number of different causes. Different color stood for different causes of death. The percentage of cardiac events, cerebral stroke, infection, alimentarytracthemorrhage and unknown were 30.30, 11.82, 16.36, 1.21 and 36.36%.

### Outcomes and risk factors among peritoneal dialysis patients

In our cohort, 1031 out of 2654 patients developed adverse events including death, drop out and roll-out. Hypoalbuminaemia was associated with adverse events which there no significant association between other baseline clinical variables and occurrence of peritonitis, except that ALB had a beneficial effect (*p* = .008) for technical survival.

Of the 2654 peritoneal dialysis patients, 330 (12.43%) patients died at the end of the study period. The major causes of death were cardiac events (30.30%), and infection (16.36%) (Figure 2).

**Figure 3.**
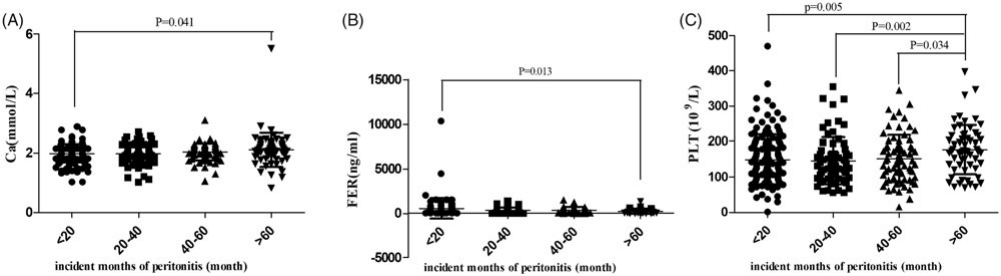
The influence of baseline clinical variable on peritonitis. *X*-axis stood for the level of clinical variables and the *Y*-axis stood for incident month of peritonitis. (A) The means of <20, 20–40, 40–60 and >60 groups were 1.98 ± 0.31, 1.99 ± 0.29, 2.03 ± 0.28 and 2.11 ± 0.57 mmol L^−1^. (B) The means of <20, 20–40, 40–60 and >60 groups were.581.63 ± 1244.71, 345.65 ± 317.12, 244.63 ± 217.26 and 271.33 ± 4303.69 ng mL^−1^. (C) The means of <20, 20–40, 40–60 and >60 groups were 581.63 ± 1244.71, 345.65 ± 317.12, 244.63 ± 217.26 and 271.33 ± 303.69 ng mL^−1^.

Total 433 patients developed peritonitis, and 126 out of these had peritonitis just once. From 2010 to 2016, the peritonitis rates were 0.09, 0.16, 0.11, 0.09, 0.08, 0.12 and 0.06 per patient-year, respectively. About 195 patients (45.03%) out of 433 patients were detected the etiological agents of peritonitis. Among these, the percent of staphylococcus aureus, Staphylococcus epidermidis, Streptococcus, Escherichia coli and Klebsiella were 13.33%, 19.49%, 13.33%, 9.74% and 4.62%, respectively. The most common etiological agent of peritonitis was Staphylococcus. By comparison, peritonitis occurred most frequently in 2011. According to the Kaplan–Meier survival analysis, there were no significant differences between baseline clinical variables and peritonitis (*p* > .05). According to the incident months of peritonitis, we divided the patients into four groups, <20, 20–40, 40–60 and 60 months, respectively. Compared with <20 months group, the level of Ca and Platelet (PLT) in >60 months group were higher, and the level of FER in >60 months group was lower (Figure 3).

## Discussion

In our study, the population of peritoneal dialysis has dramatically grown. Our study reflected that the trends on the rise in the patients on peritoneal dialysis observed were in line with those observed in Sichuan Provincial People’s Hospital which the record data of was extremely complete. The data implied that more and more ESRD patients are able to get this kind of treatment. Although there might be confouders that contribute to the rise, such as better registration, other factors such as broader coverage of medical insurance and the encouragement of government might play an important role in the increasing PD population.

In our cohort, the mortality rate has gradually decreased. However, the dialysis rates at 10 years remained at low level. Middle-aged men accounted for the majority of the proportion in our cohort. Medicine treatment and ALB more than 35 g L^−1^ were beneficial for technical survival. Consistent with other previous reports, our study showed that cardiac events were the main cause of death [16–18] and peritonitis occurring during the peritoneal dialysis must be taken seriously [19,20].

We tried to investigate the underlying reasons for peritonealitis and found that high FER level and low PLT level were both associated with peritonitis. These two indexes are associated with inflammation. Thus, inflammation or micro-inflammation might be an intervention target to lower peritonealitis.

Current literatures showed that the annual global growth rate of peritoneal dialysis is impressive reaching ∼8%. However, use of peritoneal dialysis differs dramatically between different countries [4]. Although Australia and New Zealand where the absolute number of prevalent patients on peritoneal dialysis increases has the global highest rates of peritoneal dialysis, proportion of the peritoneal dialysis population has progressively fallen. The use of peritoneal dialysis in Europe, Africa and Canada has the same trend, which is multifactorial including financial disincentives and prohibitive costs to some developing countries. On the contrary, with the financial incentives owing to government policies, Asia, especially China and Thailand, has substantial growth in peritoneal dialysis, which was consistent with our results. But population ageing, diabetes and disparity in health care provision are still challenges for these countries [4].

Nowadays, peritoneal dialysis and hemodialysis are currently two major alternatives to RRT. The choice of dialysis modality depends on quality of life, survival rates, costs, patients’ wishes and national and hospital policy [21,22]. Hemodialysis now remains the most widely dialysis mode for ESRD patients. It requires not only patients to come to dialysis units, but also a certain number of doctors and nurses to operate. Peritoneal dialysis has following advantages over hemodialysis: (1) Convenience, Peritoneal dialysis is more convenient, which means patients could dialysis at home. (2) Flexibility, patients can have dialysis at night, so that they could still work during the day. (3) Peritoneal dialysis was more cost-effective [4]. (4) Peritoneal dialysis seems to have a better outcome, for example, the renal residual function (RRF) decline delayed in patients on peritoneal dialysis not hemodialysis [23]. Thus, analyzing center characteristics, summing up experience and controlling the quality of PD are very important.

To our knowledge, this was the first largest report of peritoneal dialysis based on the registry in the Southwest of China, which provided a reference database for the further research in the future. The crux of limitation in this study was that since some centers did not attach importance to the registry and were unfamiliar with the operation, some information of patients on dialysis was missing and the registry data was not standardized in the first 2 years. So, the data of 2010–2011 may be kept skeptical, but the overall trend was valuable.

## Conclusions

In conclusion, our results, representing the first largest report of peritoneal dialysis in the Southwest of China, indicated the increasing number of patients receiving peritoneal dialysis, which will require the need for more medical resource.
